# Augmented reality advances in visualizing rare vascular anomalies of von Hippel–Lindau syndrome during neurosurgery

**DOI:** 10.1097/MS9.0000000000004136

**Published:** 2025-10-29

**Authors:** Muhammad Talha, Awais Butt, Mahnoor Fatima, Omar Abdullah Gill

**Affiliations:** a Department of Medicine, King Edward Medical University, Lahore, Pakistan; b Department of Medicine, Aziz Fatimah Medical and Dental College, Faisalabad, Pakistan; c Department of Medicine, Shaheed Ziaur Rahman Medical College, Rajshahi Medical University, Bogura, Bangladesh

Von Hippel–Lindau (VHL) syndrome is a rare genetic disorder that results in the development of soft tissue defects, such as an abnormal, nonfunctional configuration linked to hemangioblastoma or retinal vasculopathy, or vascular neoplasms. These tumors are challenging to treat because they are highly vascularized, hard to reach, and frequently complex anatomically. For the excision to be as effective as possible while preserving critical neurological function, such entities should be precisely mapped and visualized intraoperatively^[1,2]^. This is in line with the TITAN Guidelines on the need for transparency in AI use in healthcare^[3]^.

With augmented reality (AR) systems now revolutionizing the neurosurgical field by actually overlaying three-dimensional vascular maps in real-time directly into the surgical field, advances in the field now take advantage of high-definition imaging modalities like MRI and CT, which facilitate patient-specific vascular anatomical reconstructions with great detail. With molecular imaging, it becomes easier to delineate pathological vasculature for better surgical targeting and optimization of resection margins. For example, AR-assisted navigation has shown improvement in the precision of localization of vascular feeders and draining veins in hemangioblastomas related to VHL for safer tumor resection, with shorter operative time and reduced complications^[4,5]^.

Aside from that, AR platforms today integrate electromagnetic tracking, as well as deformable registration algorithms, for intraoperative vascular shift compensation, keeping accurate overlays even under dynamic surgical conditions. Multicenter validation studies have proven the reproducibility and clinical usefulness of AR guidance for vascular anomalies; they have demonstrated better surgical outcomes and neurologic preservation from various neurosurgical centers. Despite advancements, there is still limited dissemination due to expensive systems and complicated techniques, with further validation required in larger patient populations^[6]^.

Research in the future should focus on standardizing AR protocols and the seamless integration of molecular imaging into AR platforms for personalized, precision-guided neurosurgery for VHL and other rare vascular pathologies, thus overcoming the barriers. The convincing data from multicenter trials call to find out the clinical benefits and cost-effectiveness of AR-assisted vascular anomaly management, a new way of defining clinical guidelines and surgical curricula. Perhaps, these innovations may also be democratized to neurosurgical education through AR training modules^[7]^.

AR quickly modifies the surgical area for vascular anomalies in VHL syndrome, wherein balanced intricate vascular networks and highly risky surgery necessitate the utmost precision. Initial reports show great potential increases with regard to intraoperative visualization, vascular feeder localization, and complication reduction, but all have only been carried out in dedicated institutions involving very few patients. The next steps would be standardization of AR protocols so that they could be replicated at other institutions, multicenter clinical trials to prove benefits on a larger scale, and cost-effectiveness studies that could facilitate inclusion in policy. This will also have the advantage of developing scenarios in which AR-based curricula are introduced as training programs to democratize access and prepare neurosurgeons with the requisite competence to utilize these technologies effectively. This perspective intersects with literature to emphasize the fact that it is possible to make promises with AR, not by its mere technical newness but through deliberate efforts to validate, standardize, and meaningfully integration into everyday neurosurgical practice^[8,9]^.

To conclude, improved reality technology would be a great improvement in illustrating and managing complex vascular anomalies of VHL syndrome in neurosurgical operations. If further technologies and multicenter validation are applied, AR is capable of becoming an integral part of preoperative evaluation and intraoperative navigation, ultimately enhancing safety and clinical outcomes for patients.

## Data Availability

Not applicable to this study.

## Research registration unique identifying number (UIN)

No human subjects were involved as the type of research being submitted is an editorial.
